# Association of myocardial infarction and angina pectoris with obesity and biochemical indices in the South Korean population

**DOI:** 10.1038/s41598-022-17961-y

**Published:** 2022-08-12

**Authors:** Jeong Hee Chi, Bum Ju Lee

**Affiliations:** 1 Department of Computer Science and Engineering, Konkuk University, Seoul, Republic of Korea; 2 Digital Health Research Division, Korea Institute of Oriental Medicine, Deajeon, Republic of Korea

**Keywords:** Diseases, Medical research, Risk factors

## Abstract

The best obesity index for myocardial infarction or angina pectoris (MIAP) risk assessment remains controversial. Furthermore, the association between biochemical indices and these diseases is unclear. This study examined associations of obesity and biochemical indices with MIAP in the Korean population. This large-scale cross-sectional study was based on the Korea National Health and Nutrition Survey dataset from 2010 to 2019. A total of 22,509 subjects (9452 men and 13,057 women) aged ≥ 50 years were included. Participants consisted of 21,426 individuals without MIAP (men = 8869, women = 12,557) and 1083 with MIAP (men = 583, women = 500). Binary logistic regression was performed to examine the association of MIAP with obesity and biochemical indices. The prevalence of MIAP in Korean adults aged ≥ 50 years was 4.81% (6.57% among men, 3.98% among women). MIAP was more strongly associated with total cholesterol than other variables in men (adjusted OR = 0.436 [0.384–0.495], adjusted p < 0.001) and women (adjusted OR = 0.541 [0.475–0.618], adjusted p < 0.001). The waist-to-height ratio (adjusted OR = 1.325 [1.082–1.623], adjusted p = 0.007) and waist circumference (adjusted OR = 1.290 [1.072–1.553], adjusted p = 0.007) showed a significant association with MIAP in men, with no association between obesity indices and MIAP in women after adjustment. The association between biochemical indices and MIAP differed slightly according to sex. Only total cholesterol, creatinine, and platelets were associated with MIAP in both men and women.

## Introduction

Cardiovascular disease is the leading cause of mortality worldwide^1,2^. A recent 1990–2019 global cardiovascular disease-related study by the American Heart Association reported that the number of patients with cardiovascular disease has approximately doubled over the past 30 years, and the mortality rate has also been steadily increasing^1^. In addition, according to the 2021 Korea Centers for Disease Control and Prevention report titled Chronic Diseases and Issues, the mortality rate from heart disease has been increasing over the past decade, and the mortality rate of heart disease is the second highest after that of cancer^2^. Myocardial infarction and angina pectoris (MIAP) are significant causes of cardiovascular disease, and it is essential to identify risk factors for these diseases.

The known risk factors for MIAP are very diverse and are as follows: waist circumference (WC)^3,4^, waist-to-hip ratio (WHR)^5–8^, body mass index (BMI)^9,10^, waist-to-height ratio (WHtR)^11^, serum cholesterol and triglyceride levels, smoking, hypertension^4,12^, fasting blood glucose level, high-density lipoprotein cholesterol (HDL-C) level, blood pressure^4^, family history of acute myocardial infarction^13^, thickness of the carotid-artery intima media^14^, mean platelet volume^15^, abnormal glucose tolerance^16^, race and ethnicity^17^. For example, increased thickness of the carotid-artery intima media increases the risk of myocardial infarction and stroke^14^. Moderate alcohol intake (2 to 4 times a week) was related to a lower risk of myocardial infarction in both men and women^18^. Higher BMI was significantly associated with the occurrence of myocardial infarction, angina pectoris, or cardiac death^10^. Greater WC and higher WHR were independently related to the risk of myocardial infarction and death resulting from coronary heart disease^6^. Additionally, abnormal glucose tolerance was twice as common in the acute myocardial infarction group as in the normal group^16^. A greater ratio of cholesterol to HDL-C was a predictor of the first event of myocardial infarction, angina pectoris, or cardiac death^10^.

The objective of this study was to examine the association of obesity and biochemical indices with MIAP in the South Korean population. Until now, although many studies have examined associations of biochemical and obesity indices with MIAP, the best index for MIAP risk assessment among various obesity indices remains controversial. One controversial issue was that many studies have indicated that the most important indices for myocardial infarction risk assessment are WHtR^11^, WHR^5–7^, WC^3,4,6^, and BMI^9,10^ according to various major ethnic groups^7^. The other issue was that the association between various biochemical indices and acute myocardial infarction is unclear^19^ because several studies reported that triglyceride levels were associated with MIAP^4^, whereas some studies indicated that triglyceride levels were not associated with the diseases^19,20^. Therefore, in this study, we reveal the associations of MIAP with biochemical and obesity indices in a Korean population. Our findings may help clarify the risk factors for MIAP in the Korean population.

## Results

### The demographic characteristics of the subjects

Table 1 indicates the demographic characteristics for all indices. A total of 22,509 subjects aged ≥ 50 years were included in the final analysis. The final dataset consisted of 21,426 subjects without MIAP (men = 8869, women = 12,557) and 1083 subjects with MIAP (men = 583, women = 500). The prevalence of MIAP in Korean adults aged ≥ 50 years was 4.81% (6.57% for men and 3.98% for women). All other indices analyzed in this study except for ischemic heart disease (IHD) family history (p = 0.588), systolic blood pressure (SBP, p = 0.146), and BMI (p = 0.721) showed statistically significant differences between men and women. We also analyzed the difference between the non-MIAP and MIAP groups. Among the demographic factors, age, education level, occupation, and household income, but not residential area, showed significant differences between the two groups in both men and women. The average age of the MIAP group (men: 65.88 ± 0.46, women: 68.7 ± 0.42) was older than that of the non-MIAP group (men: 60.84 ± 0.12, women: 61.53 ± 0.11). The MIAP group was significantly more likely to have a lower educational level and household income and a higher unemployment rate than the non-MIAP group. In particular, the difference between the non-MIAP and MIAP groups in these indices tended to be more prominent in women than in men. Among the health behavior factors, stress showed a significant difference only in women. The prevalence of 'slight' was the highest in both the non-MIAP group and the MIAP group, but the percentage of ‘very' tended to be higher in the MIAP group than in the non-MIAP group. In addition, alcohol consumption showed significant differences in both men and women, and the MIAP group was more likely to have lower alcohol consumption than the non-MIAP group. Smoking status showed no significant difference between the two groups in both men and women. Among the preliminary health examination and disease-related indices, IHD family history, hypertension, diabetes, and hypercholesterolemia showed significant differences between the two groups in men and women, whereas hypertriglyceridemia showed no significant difference in men (p = 0.095) or women (p = 0.181). In particular, the IHD family history in the MIAP group tended to be 63.10% greater in men and 110.60% greater in women than in the non-MIAP group. Among the health examination-related indices, in men, diastolic blood pressure (DBP), height, BMI, WC, WHtR, glucose, total cholesterol, triglycerides, HDL-C, hemoglobin, hematocrit, blood urea nitrogen (BUN), creatinine, white blood cells (WBCs), red blood cells (RBCs), and platelets showed significant differences between the two groups. In women, SBP, DBP, height, BMI, WC, WHtR, glucose, total cholesterol, HDL-C, aspartate aminotransferase (AST), hemoglobin, hematocrit, BUN, creatinine, WBC, RBC, and platelet showed significant differences between the two groups. SBP was significantly different between the two groups only in women, and the MIAP group was higher than the non-MIAP group, whereas DBP was lower in the MIAP group than the non-MIAP group in men and women. The MIAP group tended to be shorter and have higher weight than the non-MIAP group, but there was no substantial difference. On the other hand, WC was more likely to be larger in the MIAP group than in the non-MIAP group. Although most biochemical indices did not differ significantly between the two groups, the MIAP group was more likely to have lower total cholesterol than the non-MIAP group in men and women.

**Table 1:** Demographic characteristics of the subjects.

Variables	Men			Women		
	Non-MIAP	MIAP	p value	Non-MIAP	MIAP	p value
Numbers	8,869	583		12,557	500	
Age (years, mean ± SE) ***	60.84 ± 0.12	65.88 ± 0.46	< 0.001	61.53 ± 0.11	68.7 ± 0.42	< 0.001
Residential areas (%(SE))**			0.369			0.250
City	76.99 (1.14)	78.84 (2.00)		78.85 (1.02)	76.38 (2.24)	
Rural	23.01 (1.14)	21.16 (2.00)		21.15 (1.02)	23.62 (2.24)	
Education (%(SE))***			< 0.001			< 0.001
≤ Elementary school	22.80 (0.60)	27.18 (1.94)		42.62 (0.61)	66.06 (2.56)	
Middle school	16.90 (0.50)	21.44 (1.91)		16.67 (0.42)	16.84 (1.98)	
High school	32.30 (0.70)	31.78 (2.26)		27.97 (0.53)	12.81 (1.89)	
≥ University	28.00 (0.70)	19.59 (1.93)		12.73 (0.44)	4.29 (0.97)	
Occupation (%(SE))***			< 0.001			< 0.001
White-collar worker	12.03 (0.47)	7.20 (1.26)		4.30 (0.22)	1.30 (0.50)	
Office worker	7.71 (0.39)	4.58 (1.14)		3.22 (0.20)	0.80 (0.40)	
Service	9.26 (0.41)	7.77 (1.82)		15.91 (0.44)	7.50 (1.30)	
Farmer or fisher	11.07 (0.62)	8.88 (1.37)		6.09 (0.39)	4.20 (0.90)	
Blue-collar worker	21.93 (0.60)	14.29 (1.96)		2.71 (0.18)	0.80 (0.50)	
Elementary occupations	9.10 (0.35)	9.68 (1.46)		13.54 (0.39)	12.00 (1.80)	
Unemployed (housewife, etc.)	28.9 (0.61)	47.60 (2.37)		54.23 (0.58)	73.50 (2.30)	
Household income (%(SE))***			< 0.001			< 0.001
Low	20.14 (0.54)	28.47 (2.02)		27.44 (0.55)	43.00 (2.70)	
Middle-low	25.35 (0.59)	27.09 (2.09)		25.09 (0.49)	27.30 (2.30)	
Middle-high	25.21 (0.59)	23.97 (2.22)		22.65 (0.48)	19.50 (2.10)	
High	29.30 (0.70)	20.46 (2.23)		24.82 (0.55)	10.20 (1.70)	
Stress (%(SE))***			0.395			0.003
Extremely	3.10 (0.21)	3.78 (0.88)		4.78 (0.23)	5.94 (1.06)	
Very	14.40 (0.46)	16.60 (1.85)		19.20 (0.42)	25.28 (2.39)	
Slightly	60.42 (0.62)	56.77 (2.36)		56.29 (0.52)	47.39 (2.69)	
Rarely	22.09 (0.50)	22.85 (1.89)		19.73 (0.41)	21.38 (1.94)	
Alcohol consumption (%(SE))***			0.016			< 0.001
Never drinker for a lifetime	6.06 (0.30)	7.38 (1.23)		25.12 (0.46)	34.21 (2.48)	
Never drinker for 1 year	14.84 (0.46)	20.67 (1.91)		20.90 (0.42)	24.53 (2.23)	
< 1 per month	9.37 (0.37)	11.35 (1.73)		23.85 (0.46)	22.55 (2.26)	
1 per month	7.76 (0.35)	6.39 (1.04)		9.71 (0.31)	5.61 (1.08)	
2–4 per month	22.67 (0.54)	19.31 (1.88)		13.16 (0.37)	7.68 (1.42)	
2–3 per week	22.73 (0.56)	20.50 (2.09)		5.17 (0.24)	4.53 (1.28)	
≥ 4 per week	16.57 (0.46)	14.40 (1.74)		2.09 (0.16)	0.89 (0.51)	
Smoking status (%(SE))***			0.069			0.291
Never	17.49 (0.51)	13.20 (1.67)		93.00 (0.29)	92.33 (1.40)	
Present	28.82 (0.62)	26.83 (2.32)		3.10 (0.20)	3.30 (0.81)	
Sometimes	2.92 (0.24)	3.36 (0.81)		0.59 (0.09)	0.07 (0.07)	
Past	50.77 (0.66)	56.61 (2.60)		3.31 (0.18)	4.30 (1.15)	
**Family history of IHD (%(SE))**			0.003			< 0.001
No	93.55 (0.34)	89.48 (1.62)		93.40 (0.30)	86.10 (1.90)	
Yes	6.45 (0.34)	10.52 (1.62)		6.60 (0.30)	13.90 (1.90)	
Hypertension (%(SE))**			< 0.001			< 0.001
No	54.40 (0.60)	43.69 (2.44)		56.93 (0.55)	30.90 (2.50)	
Yes	45.60 (0.60)	56.31 (2.44)		43.07 (0.55)	69.10 (2.50)	
Diabetes (%(SE))***			< 0.001			< 0.001
No	80.67 (0.50)	68.20 (2.30)		85.50 (0.40)	68.46 (2.45)	
Yes	19.33 (0.50)	31.80 (2.30)		14.50 (0.40)	31.54 (2.45)	
Hypercholesterolemia (%(SE))***			< 0.001			< 0.001
No	79.80 (0.50)	65.21 (2.40)		67.90 (0.50)	48.00 (2.64)	
Yes	20.20 (0.50)	34.79 (2.40)		32.10 (0.50)	52.00 (2.64)	
Hypertriglyceridemia (%(SE))***			0.095			0.181
No	78.05 (0.55)	81.85 (2.01)		86.47 (0.37)	83.99 (1.93)	
Yes	21.95 (0.55)	18.15 (2.01)		13.53 (0.37)	16.01 (1.93)	
**Blood pressure (mean ± SE)**						
SBP (mmHg)	124.12 ± 0.22	123.99 ± 0.77	0.866	123.50 ± 0.20	130.33 ± 0.92	< 0.001
DBP (mmHg)***	78.24 ± 0.14	73.39 ± 0.50	< 0.001	75.47 ± 0.11	73.76 ± 0.50	0.001
**Anthropometrics (mean ± SE)**						
Height (cm)***	167.88 ± 0.08	166.80 ± 0.30	< 0.001	154.81 ± 0.07	152.80 ± 0.28	< 0.001
Weight (kg)***	68.11 ± 0.13	68.25 ± 0.48	0.770	57.84 ± 0.10	59.03 ± 0.45	0.010
Body mass index (kg/m^2^)	24.12 ± 0.04	24.48 ± 0.13	0.009	24.12 ± 0.04	25.24 ± 0.16	< 0.001
Waist circumference (cm)***	86.22 ± 0.11	88.37 ± 0.40	< 0.001	81.67 ± 0.11	85.76 ± 0.47	< 0.001
Waist-to-height ratio***	0.51 ± 0.00	0.53 ± 0.00	< 0.001	0.53 ± 0.00	0.56 ± 0.00	< 0.001
**Biochemical indices (mean ± SE)**						
Glucose (mg/dl)***	106.72 ± 0.32	109.61 ± 1.25	0.028	101.29 ± 0.25	106.88 ± 1.37	< 0.001
Total cholesterol (mg/dl)***	190.49 ± 0.49	160.40 ± 1.69	< 0.001	201.64 ± 0.41	175.94 ± 1.97	< 0.001
Triglycerides (mg/dl)***	159.19 ± 1.71	143.08 ± 5.90	0.011	130.12 ± 0.87	141.57 ± 3.93	0.052
HDL-C (mg/dl)***	47.03 ± 0.15	45.14 ± 0.59	0.003	52.17 ± 0.14	49.33 ± 0.63	< 0.001
AST (iu/l)***	25.83 ± 0.18	26.35 ± 0.59	0.406	23.15 ± 0.11	24.86 ± 0.50	0.018
ALT (iu/l)***	25.04 ± 0.21	25.02 ± 0.68	0.982	20.24 ± 0.14	21.17 ± 0.67	0.195
Hemoglobin (g/dl)***	15.07 ± 0.02	14.52 ± 0.07	< 0.001	13.26 ± 0.01	13.12 ± 0.05	0.013
Hematocrit (%)***	44.82 ± 0.05	43.48 ± 0.18	< 0.001	40.19 ± 0.04	39.77 ± 0.15	0.012
BUN (mg/dl)***	16.15 ± 0.07	17.19 ± 0.29	0.001	15.44 ± 0.05	16.56 ± 0.25	< 0.001
Creatinine (mg/dl) ***	0.97 ± 0.01	1.06 ± 0.02	0.001	0.72 ± 0.00	0.81 ± 0.01	< 0.001
WBCs (thous/ul)***	6.50 ± 0.03	6.68 ± 0.09	0.050	5.76 ± 0.02	6.21 ± 0.09	< 0.001
RBCs (mil/ul)***	4.79 ± 0.01	4.64 ± 0.02	< 0.001	4.34 ± 0.00	4.29 ± 0.02	0.005
Platelets (thous/ul)***	241.98 ± 0.78	228.77 ± 3.47	< 0.001	257.84 ± 0.69	249.16 ± 3.46	0.015

*MIAP* myocardial infarction or angina pectoris, *IHD* ischemic heart disease, *SBP* systolic blood pressure, *DBP* diastolic blood pressure, *HDL-C* high-density lipoprotein cholesterol, *AST* aspartate aminotransferase, *ALT* alanine aminotransferase, *BUN* blood urea nitrogen, *WBC* white blood cell, *RBC* red blood cell.

*p < 0.05, **p < 0.01, ***p < 0.001. *, ** and *** indicate p values for sex differences between all men and women. Continuous data are represented as the mean ± SE (standard error). Categorical data are represented as percentages (SEs). p values were obtained from Rao-Scott chi-squared tests for categorical variables and from a general linear model for continuous variables between the group without MIAP and the group with MIAP.

### Associations of MIAP with anthropometric indices and biochemical indices

Tables 2 and 3 show the associations between MIAP and blood pressure, anthropometric indices, and biochemical indices in Korean men and women aged ≥ 50 years. Of all indices, total cholesterol showed a more strongly negative association with MIAP than all other indices in men (OR = 0.401 [0.354–0.453], p < 0.001) and women (OR = 0.472 [0.413–0.539], p < 0.001) in the crude analysis. In addition, this association was strongly maintained in men (adjusted OR = 0.436 [0.384–0.495], adjusted p < 0.001) and women (adjusted OR = 0.541 [0.475–0.618], adjusted p < 0.001) after adjusting for age, residential areas, education, occupation, household incomes, stress, alcohol consumption, smoking status, IHD family history, and BMI. In addition to total cholesterol, creatine and platelets showed significant associations with MIAP in men and women in the crude and adjusted analyses. Nevertheless, except for these three variables, the association between MIAP and all other variables showed sex differences.

**Table 2:** Associations of MIAP with blood pressure, anthropometric indices, and biochemical indices in men**.**

Variables	Crude		Adjustment	
	OR (95% CI)	p value	OR (95% CI)	p value
Age	1.720 (1.558–1.898)	< 0.001		
**Blood pressure**				
SBP	0.991 (0.897–1.096)	0.867	0.900 (0.807–1.002)	0.055
DBP	0.614 (0.551–0.685)	< 0.001	0.719 (0.638–0.809)	< 0.001
**Anthropometrics**				
Height	0.837 (0.754–0.928)	< 0.001	0.994 (0.892–1.108)	0.917
Weight	1.015 (0.919–1.121)	0.769	1.000 (0.807–1.239)	0.999
Body mass index	1.129 (1.032–1.235)	0.008		
Waist circumference	1.288 (1.172–1.415)	< 0.001	1.290 (1.072–1.553)	0.007
Waist-to-height ratio	1.376 (1.255–1.507)	< 0.001	1.325 (1.082–1.623)	0.007
**Biochemical indices**				
Glucose	1.102 (1.021–1.190)	0.013	1.068 (0.978–1.167)	0.144
Total cholesterol	0.401 (0.354–0.453)	< 0.001	0.436 (0.384–0.495)	< 0.001
Triglycerides	0.848 (0.719–0.999)	0.049	0.875 (0.733–1.045)	0.140
HDL-C	0.838 (0.740–0.949)	0.005	0.910 (0.797–1.038)	0.160
AST	1.035 (0.960–1.116)	0.370	1.055 (0.976–1.142)	0.179
ALT	0.999 (0.913–1.093)	0.982	1.045 (0.958–1.141)	0.320
Hemoglobin	0.681 (0.622–0.745)	< 0.001	0.749 (0.677–0.830)	< 0.001
Hematocrit	0.708 (0.647–0.774)	< 0.001	0.777 (0.704–0.858)	< 0.001
BUN	1.138 (1.043–1.242)	0.004	1.077 (1.000–1.160)	0.049
Creatinine	1.074 (1.011–1.141)	0.020	1.066 (1.019–1.116)	0.006
WBCs	1.096 (1.006–1.193)	0.036	1.075 (0.977–1.182)	0.136
RBCs	0.711 (0.649–0.779)	< 0.001	0.787 (0.709–0.873)	< 0.001
Platelets	0.783 (0.680–0.900)	< 0.001	0.855 (0.745–0.981)	0.025

OR and p values were obtained from the crude and adjusted analyses using complex sample binary logistic regression. Age, residential area, education, occupation, household income, stress, alcohol consumption, smoking status, family history of IHD, and BMI were used as adjustment variables. Odds ratios were estimated with 95% confidence intervals.

*MIAP* myocardial infarction or angina pectoris, *IHD* ischemic heart disease, *SBP* systolic blood pressure, *DBP* diastolic blood pressure, *HDL-C* high-density lipoprotein cholesterol, *AST* aspartate aminotransferase, *ALT* alanine aminotransferase, *BUN* blood urea nitrogen, *WBC* white blood cell, *RBC* red blood cell, *OR* odds ratio.

**Table 3:** Associations of MIAP with blood pressure, anthropometric indices, and biochemical indices in women**.**

Variables	Crude		Adjustment	
	OR (95% CI)	p value	OR (95% CI)	p value
Age	1.415 (1.296–1.545)	< 0.001		
**Blood pressure**				
SBP	0.838 (0.751–0.933)	< 0.001	1.133 (1.019–1.259)	0.022
DBP	2.076 (1.892–2.278)	0.001	0.957 (0.852–1.075)	0.460
**Anthropometrics**				
Height	0.722 (0.657–0.794)	< 0.001	1.050 (0.928–1.188)	0.440
Weight	1.141 (1.035–1.258)	0.008	1.099 (0.869–1.388)	0.431
Body mass index	1.367 (1.261–1.483)	< 0.001		
Waist circumference	1.529 (1.391–1.681)	< 0.001	1.162 (0.938–1.441)	0.170
Waist-to-height ratio	1.649 (1.509–1.803)	< 0.001	1.097 (0.872–1.380)	0.430
**Biochemical indices**				
Glucose	1.187 (1.113–1.265)	< 0.001	1.080 (0.991–1.177)	0.078
Total cholesterol	0.472 (0.413–0.539)	< 0.001	0.541 (0.475–0.618)	< 0.001
Triglycerides	1.114 (1.035–1.200)	0.004	1.050 (0.937–1.176)	0.402
HDL-C	0.783 (0.694–0.883)	< 0.001	0.970 (0.858–1.097)	0.631
AST	1.106 (1.036–1.180)	0.002	1.072 (1.001–1.149)	0.047
ALT	1.058 (0.985–1.137)	0.124	1.058 (0.980–1.142)	0.149
Hemoglobin	0.884 (0.806–0.969)	0.008	0.945 (0.856–1.042)	0.257
Hematocrit	0.876 (0.793–0.968)	0.009	0.928 (0.839–1.025)	0.142
BUN	1.217 (1.130–1.311)	< 0.001	1.063 (0.977–1.157)	0.158
Creatinine	1.154 (1.065–1.251)	< 0.001	1.107 (1.051–1.166)	< 0.001
WBCs	1.270 (1.164–1.385)	< 0.001	1.098 (0.997–1.210)	0.058
RBCs	0.854 (0.766–0.952)	0.004	0.953 (0.859–1.057)	0.361
Platelets	0.863 (0.763–0.976)	0.019	0.888 (0.791–0.998)	0.046

OR and p values were obtained from the crude and adjusted analyses using complex sample binary logistic regression. Age, residential area, education, occupation, household income, stress, alcohol consumption, smoking status, family history of IHD, and BMI were used as adjustment variables. Odds ratios were estimated with 95% confidence intervals.

*MIAP* myocardial infarction or angina pectoris, *IHD* ischemic heart disease, *SBP* systolic blood pressure, *DBP* diastolic blood pressure, *HDL-C* high-density lipoprotein cholesterol, *AST* aspartate aminotransferase, *ALT* alanine aminotransferase, *BUN* blood urea nitrogen, *WBC* white blood cell, *RBC* red blood cell, *OR* odds ratio.

Among blood pressure-related variables, in men, only DBP showed a negative association with MIAP in the crude (OR = 0.614 [0.551–0.685], p < 0.001) and adjusted analyses (adjusted OR = 0.719 [0.638–0.809], adjusted p < 0.001), whereas only SBP showed a significant association with MIAP in the crude (OR = 0.838 [0.751–0.933], p < 0.001) and adjusted (adjusted OR = 1.133 [1.019–1.259], adjusted p = 0.022) analyses in women. Of the obesity indices, WC and WHtR showed a significant association with MIAP in the crude and adjusted analyses in men. In particular, WHtR (adjusted OR = 1.325 [1.082–1.623], adjusted p = 0.007) showed a more significant association with MIAP than WC (adjusted OR = 1.290 [1.072–1.553], adjusted p = 0.007). In contrast, in women, all indices showed a significant association in the crude analysis, but there was no association between MIAP and any obesity index in the adjusted analysis. Among biochemical indices, total cholesterol, creatine, and platelets were associated with MIAP in men and women, but other variables except for these variables showed sex differences. In men, hemoglobin (adjusted OR = 0.749 [0.677–0.830], adjusted p < 0.001), hematocrit (adjusted OR = 0.777 [0.704–0.858], adjusted p < 0.001), BUN (adjusted OR = 1.077 [1.000–1.160], adjusted p = 0.049), and RBC (adjusted OR = 0.787 [0.709–0.873], adjusted p < 0.001) were associated with MIAP in the crude and adjusted analyses, whereas in women, only AST (adjusted OR = 1.072 [1.001–1.149], adjusted p = 0.047) showed an additional association with MIAP in the crude and adjusted analyses.

## Discussion

In this study, we found that after adjustment for various confounders, WC and WHtR were associated with MIAP in Korean men but not in women. Furthermore, total cholesterol, hemoglobin, hematocrit, BUN, creatinine, RBCs, and platelets were associated with diseases in men, and total cholesterol, AST, creatinine, and platelets were related to diseases in women.

Regarding obesity indices, the best obesity index for MIAP risk assessment is still a controversial issue. Yusuf et al.^7^ investigated the association between myocardial infarction and BMI, WC, hip circumference, and WHR in European, Chinese, Asian, Arab, Latin American, Black African, and mixed-race African populations. They reported that WHR was the best predictor of myocardial infarction risk in both sexes, all age groups, and all ethnic groups. Lakka et al.^3^ examined the relationship of acute myocardial infarction or angina with BMI, WHR, and WC based on Cox proportional hazards models in middle-aged Finnish men and reported that WC was a stronger risk factor for acute MIAP than BMI. Wolk et al.^9^ examined the association between BMI and acute coronary syndromes, including unstable angina pectoris and myocardial infarction, in US adults and reported that BMI and C-reactive protein were independent risk factors for coronary syndromes. Zeller et al.^25^ investigated the relationship between obesity and death after acute myocardial infarction in the French population and argued that neither WC nor BMI independently predicted death after acute myocardial infarction due to the obesity paradox, and it is important to measure both WC and BMI because of the possibility of high WC and low BMI. Pandey et al.^4^ examined the association between components of metabolic syndrome and acute myocardial infarction in Nepal and argued that WC was associated with the disease. Ke et al.^11^ analyzed the association of a history of myocardial infarction, angina pectoris, ischemic or hemorrhagic stroke, and transient ischemic attack with four obesity indices in Chinese patients with type 2 diabetes. They argued that WHtR had a higher association with the studied diseases than WC, WHR, and BMI. Hartz et al.^5^ investigated the association between obesity indices and coronary artery disease (CAD), including myocardial infarction, angina pectoris, and hypertension, and reported that WHR was significantly associated with angiographic evidence of CAD in older women. Rexrode et al.^26^ examined the relationship of WC and WHR with myocardial infarction or coronary revascularization in US male physicians. They found a modest association of WHR and WC with the risk of myocardial infarction or coronary revascularization in middle-aged and older men, even though the association disappeared after BMI adjustment. Zhang et al.^8^ assessed the association of BMI, WC, WHR, and WHtR with nonfatal or fatal myocardial infarction in Chinese women by conducting a population-based and prospective cohort study. They reported that BMI, WC, and WHtR were associated with the risk of myocardial infarction in young women, but WHR was positively related to myocardial infarction risk in both young and old women. Mørkedal et al.^27^ documented that subjects with high BMI without metabolic abnormalities did not have a significant excess risk of acute myocardial infarction. Additionally, Cao et al.^28^ have argued that high WHR increased the risk of myocardial infarction, and elevated WHR was more associated in women than in men by meta-analysis. Zhu et al.^29^ mentioned that obesity and overweight increased the incidence of acute myocardial infarction through meta-analysis. Stegger et al.^30^ reported that weight and BMI were associated with incident myocardial infarction, and low and high lean body mass could be positively related to myocardial infarction. Peters et al.^31^ showed that WHR was more associated with the risk of myocardial infarction than BMI in both men and women, and increased WC and WHR were more related to the risk of the disease in women than in men. Horvei et al.^32^ reported that WHtR and WHR have the highest association with the risk of myocardial infarction among various anthropometric indices. Our finding was consistent with the results of previous studies, indicating that WC or WHtR was highly associated with MIAP in adult men^3,11,32^. However, our results did not indicate an association between obesity indices and myocardial infarction in women, as has been mentioned in previous studies^5^. Our findings indicated that the association of WC and WHtR with the studied diseases disappeared after adjustment for confounders in women.

In terms of biochemical indices, the association between various biochemical indices and acute myocardial infarction is unclear^19^. Psaty et al.^20^ assessed the association of biochemical indices, including total cholesterol, triglycerides, low-density lipoprotein cholesterol (LDL-C), and HDL-C, with myocardial infarction, stroke, and total mortality in a follow-up study in older adults in the US. They argued that most biochemical indices were not major indicators of myocardial infarction, except for a strong association between low HDL-C and myocardial infarction. Khan et al.^19^ investigated the relationship of biochemical indices, including total cholesterol, triglycerides, LDL-C and HDL-C, with acute myocardial infarction in Saudi Arabia. They compared four groups, the ST-elevated myocardial infarction patient group, non-ST-elevated myocardial infarction patient group, chest pain group, and age- and sex-matched control groups, and found that total cholesterol, LDL-C, and HDL-C were significantly lower in the patient group than in the control groups, but triglyceride levels were not associated with myocardial infarction. Correia et al.^33^ evaluated whether triglycerides, LDL-C, and HDL-C predict in-hospital events in patients with non-ST-elevated myocardial infarction or unstable angina pectoris in a Portuguese population. They argued that HDL-C significantly predicted in-hospital events, but triglycerides and LDL-C did not predict the events. Krumholz et al.^34^ examined the association of serum cholesterol levels with hospitalization for acute myocardial infarction or angina pectoris or mortality from coronary heart disease in subjects older than 70 years in a prospective cohort study. They argued that a high cholesterol level or low HDL-C was not a risk factor for hospitalization for MIAP or coronary heart disease mortality. However, total cholesterol was significantly higher in patients with myocardial infarction than in control groups^35^. Pandey et al.^4^ tested the association between each component of metabolic syndrome and acute myocardial infarction and argued that triglyceride and fasting blood glucose levels had high predictive values for myocardial infarction. Stampfer et al.^35^ examined the association between subfractions of HDL-C and the risk of myocardial infarction in male physicians in the US. They suggested that HDL-C was associated with myocardial infarction and was a strong indicator of the risk of myocardial infarction. Kaplan et al.^36^ investigated the prediction of prognosis in patients who survived a first myocardial infarction in the US and reported that high creatinine was a powerful, independent predictor of various adverse outcomes after first myocardial infarction. Dyrbuś et al.^37^ identified risk factors for death following myocardial infarction in Poland and argued that the risk factors were increased creatinine level, low hemoglobin, old age, diabetes, and left ventricular ejection fraction. Our findings were consistent with the results of previous studies, indicating that total cholesterol was lower in myocardial infarction patients than in the normal group^19^, and triglycerides were not associated with myocardial infarction^19,20,33^. Our results showed that total cholesterol levels were lower in MIAP patients than in normal patients in both men and women. We assume that one of the reasons for this phenomenon is that patients with MIAP are prescribed medications such as statine to reduce total cholesterol levels. However, further study is needed to reveal this phenomenon.

This study has some limitations. It is difficult to determine causality from our results because of the cross-sectional nature of this study design. Our results may not apply to other ethnic groups and countries because environmental and sociodemographic features may differ according to ethnicity and country. Additionally, we did not consider drug prescription information for the treatment of myocardial infarction or angina pectoris because the information was not sufficient in our data. Despite these limitations, our study has strengths. Our findings based on a population-based study using KNHANES national representative samples support the powerful statistical results.

In conclusion, this large-scale cross-sectional study examined associations of obesity and biochemical indices with MIAP in the Korean population. We found that MIAP was significantly associated with WC and WHtR obesity indices in men but not women. Only total cholesterol, creatinine, and platelets were related to MIAP in both men and women because the association between blood profiles and MIAP differed according to sex.

## Methods

### Study population and data sources

**Figure 1:**
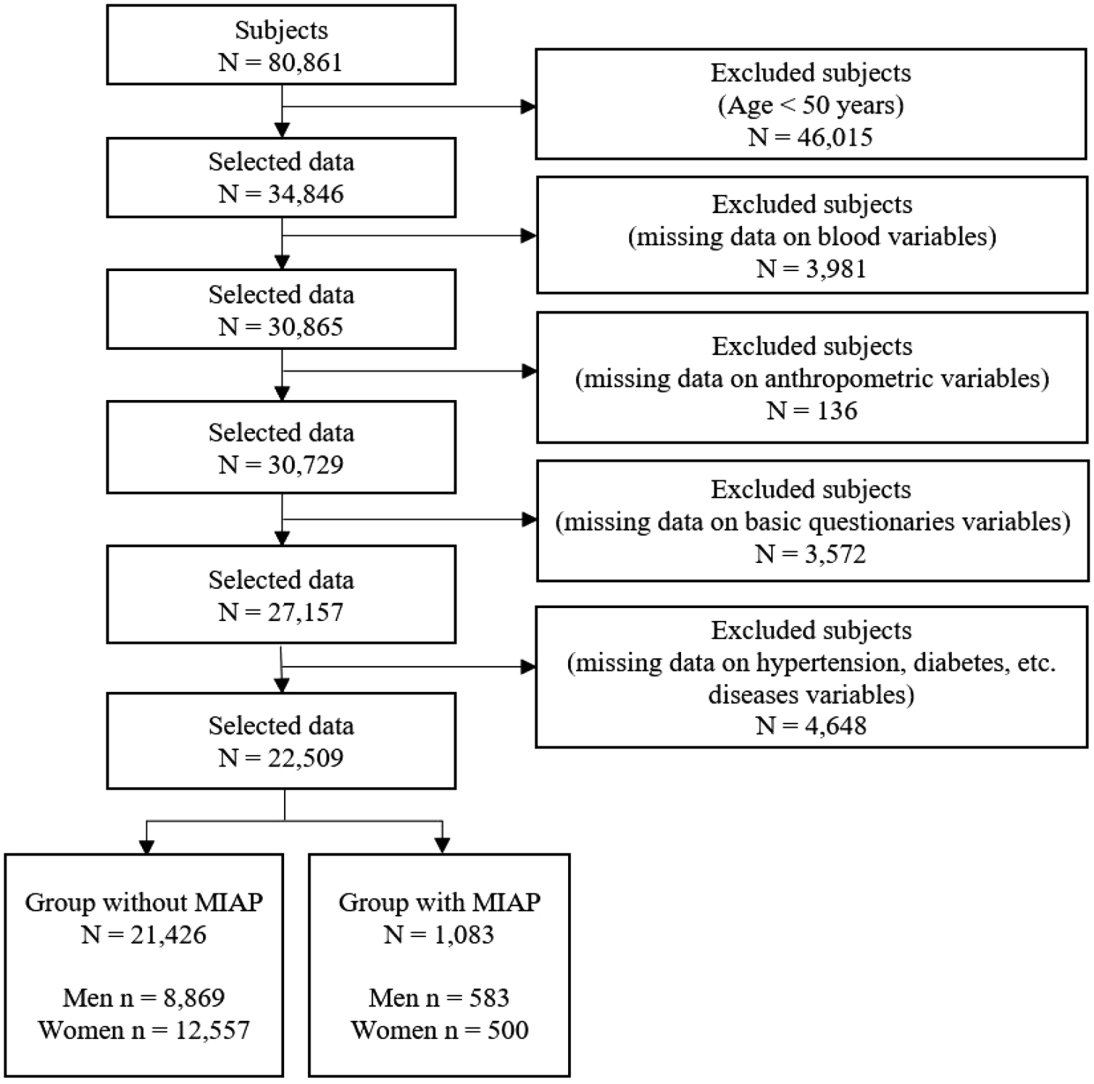
Sample selection procedure. *MIAP* myocardial infarction or angina pectoris.

The Korea National Health and Nutrition Survey (KNHANES) is a cross-sectional survey that provides reliable, nationally representative statistics on the health level, health behavior, food behaviors, and nutrition of Korean people. The survey has been conducted by the Korea Centers for Disease Control and Prevention Agency (KCDCPA) every year since 2007. We used the KNHANES dataset from 2010 to 2019, which included diagnoses of MIAP^21–24^. The KNHANES dataset used in the present study includes 80,861 subjects (men = 36,827, women = 44,034) who participated in the health survey and health examinations over the past ten years. Among the health survey variables, variables related to household surveys, education, and economic activities were collected by face-to-face interviews, and variables related to health behavior, such as smoking and alcohol consumption, were collected by a self-report method. Variables such as height, weight, and blood tests were collected through the health examination. All subjects gave their written informed consent for participation in this survey. All methods were carried out in accordance with relevant guidelines and regulations. The KNHANES datasets were approved by the KCDCPA (2010-02CON-21-C, 2011-02CON-06-C, 2012-01EXP-01-2C, 2013-07CON-03-4C, 2013-12EXP-03-5C, 2018-01-03-P-A, 2018-01-03-C-A). Additionally, this study, based on the KNHANES data, received ethics approval from the Institutional Review Board of the Korea Institute of Oriental Medicine (KIOM) (IRB No. I-2007/006-003 and I-2202/002-001). According to the inclusion and exclusion criteria, we finally selected 22,509 subjects out of 80,861 subjects. The detailed sample selection procedure is shown in Fig. 1.

### Definitions

Data regarding MIAP status were collected through the health interview and were determined by responses to two questions: “Do you have myocardial infarction diagnosed by a physician?” and “Do you have angina pectoris diagnosed by a physician?” The group with MIAP consisted of subjects who answered “yes” to at least one of the two questions, and the group without MIAP consisted of subjects who answered “no” to the questions according to the KCDCPA guidelines^24^. We determined the status of diseases such as hypertension, diabetes, hypercholesterolemia, and hypertriglyceridemia according to the guidelines from the KCDCPA. Hypertension was defined as SBP ≥ 140 mmHg, DBP ≥ 90 mmHg, or self-reported current use of antihypertensive medication. Diabetes was defined as fasting plasma glucose ≥ 126 mg/dl, glycated hemoglobin ≥ 6.5%, self-reported current use of antidiabetic medication, or doctor-diagnosed diabetes. Hypercholesterolemia was defined as fasting total cholesterol ≥ 240 mg/dl or self-reported current use of cholesterol-lowering medication. Hypertriglyceridemia was defined as fasting triglyceride levels ≥ 200 mg/dl.

### Measurement

We considered demographic factors, health behavior factors, and health examination factors as potential risk factors for MIAP. Among the variables collected through the health interviews, we used sex, age, residential area, education, occupation, and household income as demographic factors. Here, household income is grouped into quartiles based on the average monthly household income. We used stress, smoking status, and alcohol consumption as health behavior factors. These variables were collected through a self-reported method. Smoking status was grouped into four categories depending on the current smoking status. Alcohol consumption was categorized into seven levels based on the frequency of alcohol consumption during the past year. Stress was categorized into four kinds according to the usual stress perception level. Family history of IHD was collected through a preliminary survey for health examination and assessed by asking the following questions: “Do you have a parent, brother, or sister with ischemic heart disease diagnosed by a physician?”.

In the health examination, data on anthropometric indices such as height, weight, WC, blood pressure, and biochemical indices were collected according to standard protocols. BMI was calculated as weight (kg) divided by the square of height (m^2^). WHtR was calculated as WC divided by height. Blood pressure was measured three times using a standard mercury sphygmomanometer (Baumanometer Wall Unit 33(0850)/USA) at the position where the heart and arm were at the same height after resting for 5 min in a seated state and then calculated as the average value of the second and third measures. Blood samples were obtained after fasting for at least 8 h. Glucose, total cholesterol, triglycerides, HDL-C, AST, ALT, and BUN were analyzed using a Hitachi Automatic Analyzer 7600 (Hitachi/Japan) in the 2011–2012 surveys, a Hitachi Automatic Analyzer 7600-210 (Hitachi/Japan) in the 2013–2018 surveys, and a Labospect008AS (Hitachi/Japan) in the 2019 survey; creatinine was analyzed using a Hitachi Automatic Analyzer 7600 (Hitachi/Japan) in the 2011–2012 surveys, a Hitachi Automatic Analyzer 7600-210 (Hitachi/Japan) in the 2013–2018 surveys, and a Cobas (Roche, Germany) in 2019 survey; and hematocrit, hemoglobin, white blood cells (WBCs), red blood cells (RBCs), and platelets were analyzed using a XE-2100D (Sysmex, Japan) in 2010–2014 surveys and a XN-9000 (Sysmex, Japan) in 2015–2019 surveys. The KNHANES provides more detailed information on the measurement methods and equipment used during the health examination^21–24^.

### Statistical analysis

KNHANES data were collected using a multistage stratified cluster sampling design. To obtain results representing the Korean population, we performed complex sampling that applied stratification, clustering, and weights considering health questionnaires and health examinations. All statistical analyses were performed by complex sample data analysis using SPSS 21 for Windows (SPSS, Inc., Chicago, IL, USA). Continuous variables are presented as the mean ± standard error (SE), and categorical variables are presented as percentages (SEs). To compare sex differences in general characteristics and lifestyle characteristics, we performed Rao-Scott chi-squared tests for categorical variables and t tests using a general linear model for continuous variables. We also compared the differences between the group without MIAP and the group with MIAP using the same method. Table 1 shows the demographic characteristics of all indices used in the present study. A binary logistic regression model was used to analyze the associations of MIAP with obesity and biochemical indices. This model was adjusted for age, residential areas, education, occupation, household income, stress, alcohol consumption, smoking status, family history of IHD, and BMI. Odds ratios are presented with 95% confidence intervals.

## Data Availability

Data used in this study are available from KNHANES (KCDC). Anyone can freely access the data (https://knhanes.kdca.go.kr/knhanes/sub03/sub03_02_05.do).
